# S-Nitroso-*N*-Acetyl-D-Penicillamine Modified Hyperbranched Polyamidoamine for High-Capacity Nitric Oxide Storage and Release

**DOI:** 10.3390/bioengineering7010009

**Published:** 2020-01-10

**Authors:** Sean P. Hopkins, Megan C. Frost

**Affiliations:** Department of Biomedical Engineering, Michigan Technological University, 1400 Townsend Dr., Houghton, MI 49931, USA; sphopkins@mtu.edu

**Keywords:** nitric oxide, hyperbranched polymer, polyamidoamine, S-nitroso-*N*-acetyl-D-penicillamine, SNAP

## Abstract

Synthetic nitric oxide (NO)-donating materials have been shown to have many beneficial effects when incorporated into biomedical materials. When released in the correct dosage, NO has been shown to increase the biocompatibility of blood and tissue contacting materials, but materials are often limited in the amount of NO that can be administered over a period of time. To address this, hyperbranched polyamidoamine (HPAMAM) was modified with the S-nitrosothiol, S-nitroso-*N*-acetyl-D-penicillamine, and nitrosated to form a controlled, high-capacity NO-donating compound (SNAP-HPAMAM). This compound has the potential of modifying polymers to release NO over long periods of time by being blended into a variety of base polymers. Nitric oxide release was triggered by photoinitiation and through passive ion-mediated release seen under physiological conditions. A material that delivers the beneficial dose of NO over a long period of time would be able to greatly increase the biocompatibility of long-term implantable devices. Structural analysis of a generation 2 HPAMAM molecule was done through Fourier transform infrared spectroscopy (FTIR), ^1^H nuclear magnetic resonance spectroscopy (NMR), and matrix assisted laser desorption ionization, time of flight (MALDI-TOF) mass spectrometry. The NO capacity of the finalized generation 2 SNAP-HPAMAM compound was approximately 1.90 ± 0.116 µmol NO/mg. Quantification of the functional groups in the compound proved that an average of 6.40 ± 0.309 reactive primary amine sites were present compared to the 8 reactive sites on a perfectly synthesized generation 2 dendrimer. There is a substantial advantage of using the hyper-branched HPAMAM over purified dendrimers in terms of reduced labor and expense while still providing a high-capacity NO donor that can be blended into different polymer matrices.

## 1. Introduction

Nitric oxide (NO) has been proven to be an important molecule with multiple functions within the human body such as maintaining vascular health, mediating inflammatory response, and preventing bacterial adhesion [1,2,3]. To further the advancement of biomedical devices and sensors while ensuring reliable performance, the appropriate release of NO may be able to mediate unwanted foreign body response toward implantable devices. For patients that are in critical condition where a critical analyte such as lactate, glucose or oxygen is continually monitored, implanted sensors must be able to accurately measure the analyte and report real-time changes in physiological levels. Due to the foreign body response, implanted sensors often lose their functionality over unpredictable periods of time. NO has the ability to reduce this unwanted inflammatory response and increase the longevity of implantable medical devices. The two most popular classes of NO donors to be applied to biomedical materials are S-nitrosothiols and *N*-diazeniumdiolates [4,5]. These types of molecules have been covalently attached or blended within a wide variety of polymers and macromolecules to provide stable, NO-releasing materials. The release mechanisms of these and other NO donors have been well characterized and have their own specific uses for in vivo applications [6]. Long-term NO release at a physiological level is necessary for keeping implantable devices free from unwanted inflammatory response, indicating the potential utility of the slower and more controlled release mechanism of S-nitrosothiol chemistry. Using an NO donor with the potential for a high NO reservoir will allow polymers to contain the total load of NO required for these long-term situations.

Dendrimers are highly branched, symmetrical macromolecules that can have a wide array of surface chemistry characteristics. They start with a core molecule and can progress in size and functionality with stepwise reactions completed in separate batch processes. Several groups of researchers pioneered the development of dendritic type macromolecules to eventually lead to the large field of dendrimer research that is seen today [7,8,9]. A similar class of macromolecule to dendrimers are hyperbranched polymers. Hyperbranched polymers do not have the perfect symmetry that is seen with dendrimers and are usually a large array of varying sized molecules as there are no purification or chromatography steps during the processing. Dendrimers and hyperbranched molecules that are appropriately derivatized both have the ability to contain a large reservoir of NO due to the high level of surface functional groups they contain. Dendrimers have been widely used in medicine and are continuing to grow in popularity due to their molecular storage capabilities and high functionality [10]. They are used in a variety of medical applications such as cancer treatments, gene therapy, and precise drug delivery [10,11,12,13]. A popular class of dendrimer that is widely used in research is polyamidoamine (PAMAM). PAMAM dendrimer and hyperbranched polymer cytotoxicity has been previously investigated and found that the main mechanism for which these molecules decrease cell viability is through cationic disruption of cell plasma membranes [14,15]. By modifying the primary amine sites with SNAP functional groups, PAMAM dendrimers have been shown to have significantly reduced negative effects on the cells they interact with [16].

Synthesis of NO-releasing dendrimers have been reported by Stasko et al. that demonstrate the high NO storage capability of this type of molecular architecture [17]. The authors were able to fully characterize 64 armed dendrimers with the ability to deliver large, precise amounts of NO and were shown to be highly antimicrobial [18]. These types of macromolecules have then been applied to biomaterials such as polyurethane nanofibers as potential wound dressing as NO has been also to promote reendothelialization as well [19]. While this type of NO-releasing molecule is excellent for targeted therapeutics, it would be costly to integrate a large quantity of these dendrimers into polymer matrices. The synthetic route for creating high generation dendrimers is labor and time intensive due to the extensive purification processes required during the synthetic procedure. An alternative macromolecule to use for blending within polymer matrices are hyperbranched polymers. Hyperbranched polymers are similar to dendrimers but contain defects within the structure due to eliminating these purification processing steps during synthesis [20]. Hyperbranched polyamidoamine (HPAMAM) molecules are able to be synthesized in a simple and economical method with a high yield of product that is obtained over a shorter period of time compared to pure PAMAM dendrimers. The importance of the compound is stressed in the average number of functional groups present more than the dendritic structure when considering high-capacity, blendable NO-donating moieties.

NO-releasing hyperbranched polyethers have been previously synthesized incorporating *N*-diazeniumdiolate NO donors, but have shown poor NO addition efficiency, obtaining a surprisingly small NO reservoir of 0.43 µmol/mg [21]. *N*-diazeniumdiolate molecules also do not have the controllability in NO release seen with SNAP based NO donors as its release mechanism is triggered when put in physiological pH and temperature [22]. The purpose of creating a SNAP-HPAMAM compound is to easily synthesize an additive that contains a large reservoir of NO that can be blended with other polymers while still maintaining all of the beneficial properties of a SNAP based NO donor, giving the option of either having a controlled or passive release of NO.

Herein, HPAMAM was modified with the thiol, *N*-acetyl-D-penicillamine, and nitrosated to form a controlled NO-donating compound, SNAP-HPAMAM, capable of being blended within a variety of polymers. This compound was triggered to release NO by photoinitiation and ion-mediated NO release. Structural analysis of a generation 2 HPAMAM molecule was accomplished through FTIR, ^1^H NMR, and MALDI-TOF mass spectroscopy. The NO capacity of the molecule was approximately 1.90 µmol NO/mg. Quantification of the functional groups in the compound proved that an average of 6.40 reactive primary amine sites per molecule were present compared to the 8 reactive sites on a perfectly synthesized generation 2 dendrimer.

## 2. Materials and Methods

### 2.1. Materials

Ethylenediamine, methyl acrylate, methanol, ethanol, toluene, pyridine, acetic anhydride, chloroform, hexanes, hydrochloric acid, magnesium sulfate, α-Cyano-4-hydroxycinnamic acid, Lugol’s iodine, glacial acetic acid, *N*-acetyl-D-penicillamine, and Ellman’s reagent were all purchased from Sigma Aldrich (St. Louis, MO, USA). ATTO-TAG^TM^ FQ reagent was purchased from Invitrogen (Grand Island, NY, USA). Tert-butyl nitrite (90% technical grade, Acros Organics) was purchased from Fisher Scientific. Polyvinyl chloride (PVC) (M_W_ 233,000, M_n_ = 99,000) was purchased from Sigma Aldrich (St. Louis, MO, USA).

### 2.2. Synthesis of HPAMAM

Generation 2 HPAMAM molecules were synthesized following a modified multistep procedure seen with dendrimer synthesis [23]. The core molecule to initiate the hyperbranched polymer was 83 mmol of ethylenediamine (EDA), which was dissolved in 250 mL of methanol and cooled in an ice bath. Then, 340 mmol of methyl acrylate (MA) were added drop wise to the stirring solution of EDA and methanol. The solution was then allowed to stir at room temperature for 48 h. The methanol and unreacted MA were removed by rotary evaporation at 45 °C to yield a four-armed ester terminated hyperbranched polymer. Then, 742 mmol of EDA was dissolved in 100 mL of methanol in a separate container and chilled in an ice bath. This solution was then added drop wise to the stirring ester terminated hyperbranched polymer and allowed to stir at room temperature for 72 h. Rotary evaporation was then done to remove most of the unreacted EDA and methanol at 45 °C. Excess EDA was removed by adding a toluene and methanol mixture with a ratio of 9:1 (v/v) respectively at its temperature azeotrope to yield a four-armed amine terminated molecule (generation 0 HPAMAM).

The same reaction process was then repeated where MA in two times molar excess is dissolved in 100 mL of methanol and added drop wise to the chilled generation 0 HPAMAM and allowed to stir for 48 h at room temperature. Repeating the EDA reaction step and removal method results in an eight-armed amine terminated molecule (generation 2 HPAMAM). The solution becomes noticeably more viscous and amber in color after each step. 

### 2.3. Synthesis of NAP-Thiolactone

The procedure to synthesize a self-protected *N*-acetyl-D-penicillamine (NAP) thiolactone was done according to a modified procedure developed by Moynihan and Robert [24]. In total, 5 g of *N*-acetyl-D-penicillamine was dissolved in 10 mL of pyridine in a round bottom flask, while 10 mL of acetic anhydride and 10 mL of pyridine were combined in a separate beaker. The solutions were both cooled in an ice bath for 1 h and then the acetic anhydride/pyridine solution was added to the round bottom flask. The mixture was allowed to stir at room temperature for 24 h where the solution will change to a light red color. The solution was rotary evaporated at 45 °C until the solution stopped boiling. The temperature was then increased to 60 °C until most of the solvent was evaporated to obtain an amber colored viscous liquid. The resulting product was then dissolved in 20 mL of chloroform and washed and extracted three times with 20 mL of 1M HCl. MgSO_4_ was added to the chloroform solution to remove any water and was then removed by filtration. The chloroform was removed by rotary evaporation at room temperature and the resultant crystals were collected and rinsed with hexanes and filtered. Light yellow/white colored crystals were recovered and vacuum dried.

### 2.4. Nitrosation of S-Nitrosothiol Modified HPAMAM

There are two methods that can be employed to nitrosate HPAMAM. Acidified nitrite was added to the thiol terminated hyperbranched molecule to form the S-nitrosothiol. The synthesized generation 2 HPAMAM (400 mg) was dissolved in 5 mL of deionized water. Then, 3.00 mmol (519 mg) of NAP-thiolactone was added to the stirring solution and was allowed to react for 24 h to form a NAP-HPAMAM compound. An additional 5 mL of deionized water may need to be added to ensure all of the NAP-thiolactone is dissolved. The NAP-HPAMAM solution that is formed was mixed with an equal volumetric amount of 1M hydrochloric acid and chilled in ice for one hour. An abundance of sodium nitrite (230 mg, 3.33 mmol) was then added to the solution and allowed to react at 0 °C for 45 min. As the reaction occurs, the color will change from clear/light yellow to a dark green. The solution was then rotary evaporated at room temperature until most of the solvent is removed. The resulting SNAP-HPAMAM was then dissolved in chilled ethanol and mixed thoroughly. The ethanol solution was filtered of any excess sodium nitrite or unreacted precipitates using 0.22 µm syringe filters. The SNAP-HPAMAM ethanol solution was rotary evaporated at room temperature until a green viscous precipitate is left. This precipitate was then placed in a vacuum oven at room temperature until the green viscous product is completely dried. The resulting crystalline product can then be recovered and stored. Shielding the compound from light was maintained during all processing steps whenever possible.

**Figure 1:**
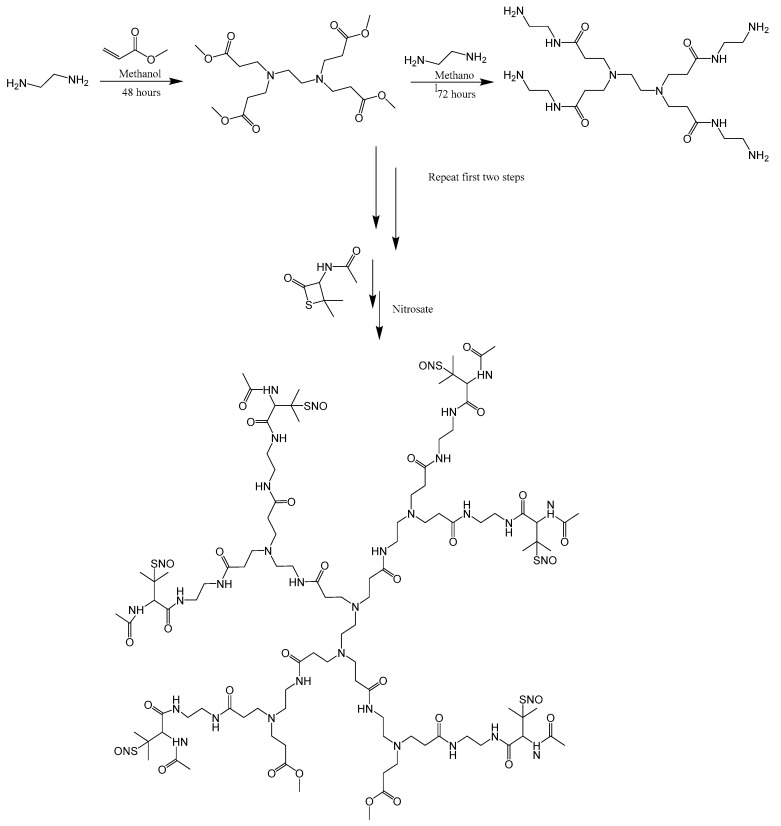
Illustration of the synthetic scheme of generation 2 SNAP-HPAMAM.

Alternatively, tert-butyl nitrite was added to the NAP-HPAMAM to form the S-nitrosothiol. Generation 2 HPAMAM (400 mg) is dissolved in 5 mL of methanol and the same amount of NAP-thiolactone is added as was previously mentioned. The solution was then allowed to react for 24 h. Then, 1 M HCl was added to the NAP-HPAMAM until the pH of the solution is approximately 5.0. The resulting NAP-HPAMAM methanol solution is then reacted with tert-butyl nitrite in excess (500 µL, 4.84 mmol). Before adding the tert-butyl nitrite, it was first cleaned by using 30 mM cyclam (1,4,8,11-tetraazacyclotetradecane) to chelate any metal ions present that could cause S-nitrosothiol (RSNO) degradation. After the addition of the cleaned tert-butyl nitrite to the NAP-HPAMAM, the solution color changed from clear/light yellow to a deep green color over the course of an hour. To remove the excess solvent, it was rotary evaporated at 35 °C and then vacuum dried at room temperature to obtain a crystalline product. For situations where your compound would need to be kept in the aqueous phase during reacting or blending with other materials, utilizing the acidified sodium nitrite method is preferred. Using tert-butyl nitrite to nitrosate the NAP-HPAMAM is the preferred method for when working with organic solvents. The reaction scheme for SNAP-HPAMAM is illustrated in Figure 1.

In order to cast PVC films containing SNAP-HPAMAH, 2 mg of SNAP-HPAMAM were dissolved in a solution containing 200 mg of PVC in 10 mL of DMAC. Films were formed by casting 0.5 mL of this solution into 10 mm Teflon ring molds. The molds were then protected from light and solvent allowed to evaporate, leaving homogenous PVC films that were approximately 100 m thick.

### 2.5. Nitric Oxide Release

Nitric oxide release from SNAP-HPAMAM was directly measured by the chemiluminescent reaction of NO with ozone using a Sievers 280i Nitric Oxide Analyzer (GE Instruments, Boulder, CO, USA). This was used to determine the variation of NO flux from polymer films containing SNAP-HPAMAM with light and for determining the total NO capacity of SNAP-HPAMAM. SNAP-HPAMAM was cast into films using polyvinyl chloride (PVC) as the base polymer. For determining photoinitiated NO release, the films were placed in a two-armed amber glass sample holder 5 cm above a mounted 460 nm blue VAOL-5GSBY4 light emitting diode (LED) obtained from Mouser Electronics Inc. (Mansfield, TX, USA). The LED was used to trigger the NO release at a variety of voltage levels at 130 Ω to show the controllability of SNAP based NO donors.

NO capacity quantification was done by tri-iodide reduction following the procedure from Yang et al. [25]. An I_3_^−^ solution was first made by creating a 3% by weight iodine solution and mixing it with acetic acid in a 2:7 ratio by volume respectively. A recorded weight of SNAP-HPAMAM was first pre-treated with a 5% acidified sulfanilamide solution to react with any unreacted sodium nitrite that could still be present from the nitrosation step. After allowing the I_3_^−^ solution to stir for 30 min, it was added to the measured SNAP-HPAMAM while being stirred at room temperature.

### 2.6. Material Characterization

FTIR (Spectrum One, Perkin Elmer), NMR (Varian 400 MHz), and MALDI-TOF (Bruker Microflex LRF) mass spectroscopy were completed to obtain the general structure of the HPAMAM and NAP-HPAMAM molecules being synthesized. Quantification of primary amines was done using by fluorescent tagging each site with ATTO-TAG FQ. Excitation was at 450 nm while the emission wavelength was at 550 nm. After each free primary amine site was reacted with NAP-thiolactone, Ellman’s test for free thiols was done to quantify the conversion of amines to thiols within the hyperbranched structures. Optical absorbance was taken at 412 nm using a 96-well plate reader. The quantified results were then used to further verify the hypothesized structures obtained from the FTIR and MALDI-TOF data.

## 3. Results and Discussion

### 3.1. Synthesis of Generation 2 SNAP-HPAMAM

Synthesis of NO-releasing PAMAM dendrimers have been previously reported utilizing both RSNOs and diazeniumdiolates [17,26]. Utilizing the high functionality of these dendridic molecules were then demonstrated to be potent antibacterial agents due to this high NO reservoir [27]. By creating a similar system with hyperbranched functionality instead of perfect dendrimer architecture, NO-releasing HPAMAM was synthesized as a high-capacity, blendable NO donor. A popular method to synthesize hyperbranched polymers is through a “one pot” synthesis route where AB_x_ monomers are polymerized together in a single polycondensation reaction [28]. However, excess crosslinking and side reactions can occur using this method, leading to a less pure final product. In the synthesis described in this study, a more complex multi-step procedure is used to form a more uniform hyperbranched polymer system. Compared to perfectly synthesized dendrimers which contain eight amine groups/molecule, the synthesized hyperbranched polymers have overall less functionality (6.40 amines/molecule) and contain unreacted or incomplete branching points in the molecular architecture. The previously used method of conjugating the primary amine end groups of the dendrimers with SNAP was then followed to modify HPAMAM to form SNAP-HPAMAM. After lyophilization, the resulting dark green, viscous liquid could then be incorporated into a variety of polymers. This hyperbranched NO donor can be readily synthesized without arduous purifications steps and then be used as an additive for a wide variety of polymers by blending it into the desired material. Additionally, NO release can be tuned by controlling the water uptake of the base polymer, the amount of SNAP-HPAMAM included and the thickness of polymer coating used for different applications. This provides for a wide range of materials to be developed that can posses tuned NO release properties and tuned mechanical properties depending on the need for the specific application. 

### 3.2. NO Release

#### 3.2.1. Photoinitiated NO Release

**Figure 2:**
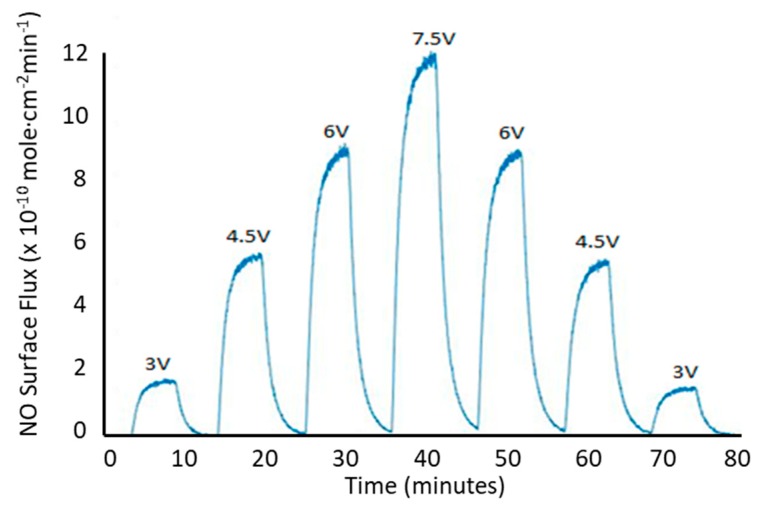
Nitric oxide (NO) release profile of SNAP-HPAMAM blended in polyvinyl chloride (PVC) using photoinitiation using a 470 nm LED and a 130 Ω resistor and different applied voltages as noted on the face of the graph.

One mechanism for releasing NO from RSNOs is via homolytic cleavage of the sulfur–nitrogen bond through the administration of light. In the dark, the SNAP-HPAMAM material is stable while at room temperature (i.e., it does not release NO), but can have a controlled and stable release of NO when irradiated with different levels of light. The stability translates to the NO release profiles seen in Figure 2, where SNAP-HPAMAM was distributed evenly (2 mg of SNAP-HPAMAM per film) into PVC thin films. The control in NO release seen with photoinitiated release at various intensities of light using a 470 nm wavelength LED at 130 Ω. Voltages are adjusted to increase or decrease the drive current to the LED, which modulates the level of light produced and the then turned off to demonstrate the time to takes to reach a normal baseline release. Once the peak level of NO release is reached for each LED illumination step, a steady state level of NO release is achieved. One benefit of having a controllable NO-releasing material is the application for in vitro testing. Different cell types interact with specific levels of NO in different ways. Most in vitro testing of NO administration is usually done by adding a solution of *N*-diazeniumdiolates NO donor directly to cell media. This method only gives a short-lived burst of NO before being diminished. Using the light-sensitive properties of RSNOs, an in vitro cell culture setup could have a tunable controlled level of NO introduced to the cells over specific periods of time.

#### 3.2.2. Ion-Mediated NO Release

**Figure 3:**
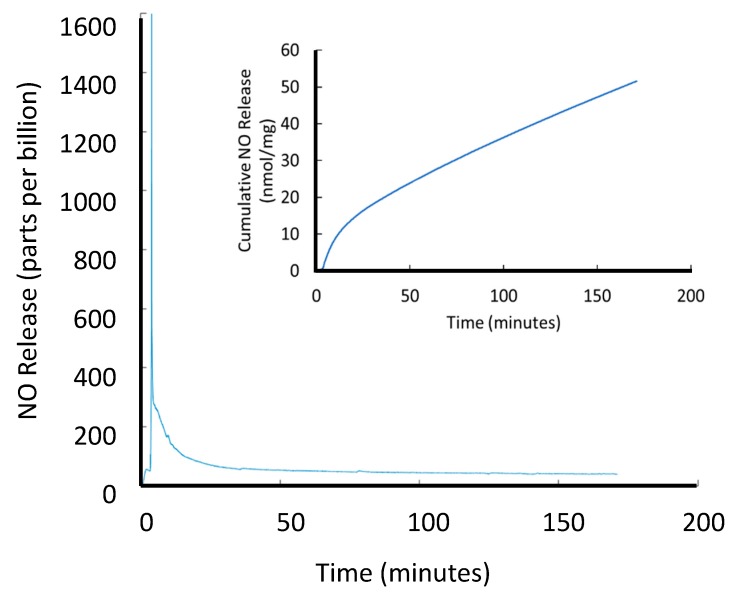
Passive NO release of 2 mL of SNAP-HPAMAM solution (1 mg/mL) in phosphate buffered saline (PBS) at 37 °C.

The SNAP-HPAMAM was also tested for its passive release from transition metal ions that are ubiquitously present in phosphate buffered saline (PBS). Certain transition metal ions such as copper, ascorbate, and iron have been previously shown to catalytically release NO from SNAP at high rates [4]. Using the SNAP-HPAMAM that was nitrosated with the acidified sodium nitrite method, multiple ion-mediated release pathways were tested. To observe the physiological stability, SNAP-HPAMAM was placed in PBS at 37 °C at a concentration of 1 mg per mL to give a representative NO release profile and is shown in Figure 3. In aqueous conditions, SNAP-HPAMAM tends to swell and dissolve readily. This explains the large burst of NO release seen once the PBS is added, until it eventually reaches a steady state.

**Figure 4:**
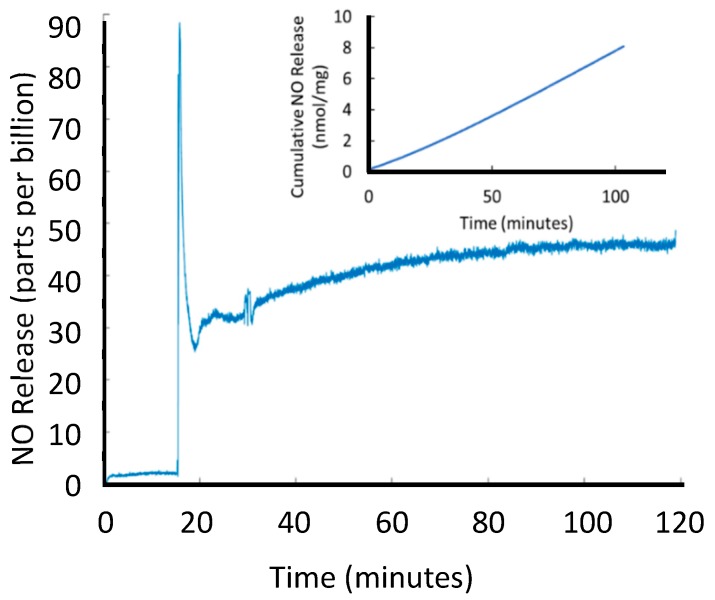
Passive NO release of SNAP-HPAMAM (2 mg per film) encapsulated in PVC in 2 mL of PBS at 37 °C.

SNAP-HPAMAM does not release NO when blended into films with other hydrophobic polymers that reduce or eliminate the diffusion of ions to the RSNO groups [4,29,30,31,32,33]. This can be used as a means to tune NO release by controlling ion diffusion and elongating the NO release from the material compared to directly exposing SNAP-HPAMAM to a solution. Figure 4 shows how this method increases the longevity of the NO donor when SNAP-HPAMAM was blended with PVC dissolved in *N,N*-dimethylacetamide and cast into 10 mm diameter films. If a hydrophilic polymer like polyvinyl alcohol was used instead, it would allow the uptake of water facilitate the diffusion of SNAP-HPAMAM into the surrounding area as it swells. This would result in a different NO release profile of the composite material. Other hydrophobic polymers like polydimethylsiloxane (PDMS) completely prevent ion diffusion into the polymer and would be suitable for situations where only thermal degradation of the RSNO is required. PVC gives the option of using ion-mediated NO release from the SNAP functional group while still providing a range of tuned release mechanism that can utilize ion-mediated decomposition, photoinitiated decomposition and/or thermal mediated NO release. A characteristic seen with blending SNAP-HPAMAM within PVC is that the hyperbranched molecule acts like a plasticizer for the PVC. Unplasticized PVC tends to be quite brittle but still manageable when cast into thin films. SNAP-HPAMAM containing PVC is less brittle than pure PVC and has more elastic properties when the concentration within the polymer matrix is high enough. Hyperbranched polyesters have been known to be suitable, safe plasticizers in PVC [34]. The unreacted, defected ester branches seen in HPAMAM are most likely providing a similar plasticizer property observed in the hyperbranched polyester blends.

### 3.3. Material Characterization

#### 3.3.1. FTIR

**Figure 5:**
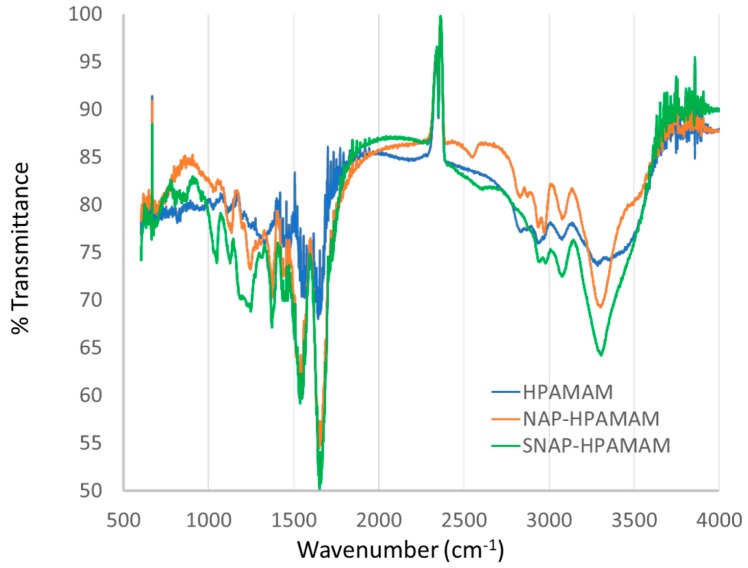
FTIR analysis of HPAMAM (blue), NAP-HPAMAM (orange), and SNAP-HPAMAM (green) compounds, the double spike that appears near 2400 cm^−1^ is an instrument artifact and used to align spectra.

Identification of the important functional groups of the HPAMAM compound was achieved with FTIR as seen in Figure 5. The presence of primary amines seen in the HPAMAM spectra around the 3300 cm^−1^ range (N-H stretching) and 1620 cm^−1^ (N-H bending), which then disappears once the attachment of NAP-thiolactone is added because it reacts with primary amine groups. An important note of transition from NAP-HPAMAM to SNAP-HPAMAM is the disappearance of the thiol peak at 2550 cm^−1^ upon nitrosated, showing an efficient addition of the NO group to the thiol moiety. The NO peak is difficult to see due to the presence of the large number of amides in the structure, which contain carbonyl groups at roughly 1500 cm^−1^. Nitroso peaks are also commonly seen in this range.

#### 3.3.2. NMR

NMR was also done on the base HPAMAM compound to verify the initial synthesis procedure is producing the correct hyperbranched structure. The important characteristic functional groups seen in PAMAM dendrimers were classified within the NMR spectrum. ^1^H NMR (400 MHz, CDCl_3_): δ 7.47 (7H, CONHCH_2_, t), 3.41 (4H, COOCH_3_, s) 3.23 (14H, NHCH_2_CH_2_, q), 2.70 (13H, CH_3_CH_2_N, t), 2.63 (11H, CH_2_CH_2_NH_2_, quin), 2.31 (19H, CH_2_CH_2_CO, t), 1.36 (22H, CH_2_NH_2_, s). The presence of unreacted ester groups and non-amide containing branches are present, but the primary amine groups remain abundant in the overall hyperbranched structure.

#### 3.3.3. Quantification of Thiols and Primary Amines

Quantification of the primary amine groups was done by attaching the fluorescent tag ATTO-TAG FQ (3-(2-furoyl) quinoline-2-carboxaldehyde) the HPAMAM molecule following the procedure developed by Liu et al. [35]. The free primary amine content present in the compound was calculated to be 4.480 ± 0.216 µmol/mg, which gives an average of 6.40 ± 0.309 amines per HPAMAM molecule. A perfectly synthesized generation 2 PAMAM dendrimer would contain 8 primary amines per molecule. Using this hyperbranched synthetic route, there was not a large loss of amine functionality in the compound.

Ellman’s test for free thiols was then used to quantify the thiol content present after reaction with the NAP-thiolactone compound to the free primary amines following a modified protocol developed by George Ellman [36]. PBS (pH = 7.4) was used in place of Tris-buffer for the procedure preventing excess unreacted NAP-thiolactone from reacting with the primary amine functional groups present in the Tris-buffer, thereby eliminating free thiol groups present other than those directly attached to the hyperbranched polymer. The free thiols content was determined to be 2.74 ± 0.298 µmol/mg.

#### 3.3.4. Determination of NO Loading

Total NO loading of SNAP-HPAMAM was determined through tri-iodide reduction and was compared to the amine and thiol quantification values. A perfectly synthesized eight-armed SNAP derivatized PAMAM dendrimer has the theoretical loading of 5.594 µmol of NO per mg. Hyperbranched polymers contain defects within the structure, so based on 6.40 primary amines/HPAMAM compared to eight primary amines/perfect dendrimer, the theoretical loading of NO should be 4.795 µmol of NO per mg. The synthesized SNAP-HPAMAM demonstrated a capacity of 1.901 ± 0.116 µmol of NO/mg. Comparing the amount of NO released with the theoretical max shows a 42.4% conversion of HPAMAM to SNAP-HPAMAM. The majority of the reduced NO release capacity occurs after the addition of the parent thiol compound to the HPAMAM (the theoretical thiol content should be 4.795 µmol/mg but was experimentally measured to be 2.74 ± 0.298 µmol/mg). The conversion percentage from the thiol content to measured NO (2.74 ± 0.298 µmol/mg theoretical and 1.901 ± 0.116 µmol of NO/mg measured) was 69%. The loss in NO observe is most likely due to the processing steps for isolating the material. The prolonged vacuum drying and rotary evaporating during the synthesis would account for part of the NO loss.

#### 3.3.5. MALDI-TOF

**Figure 6:**
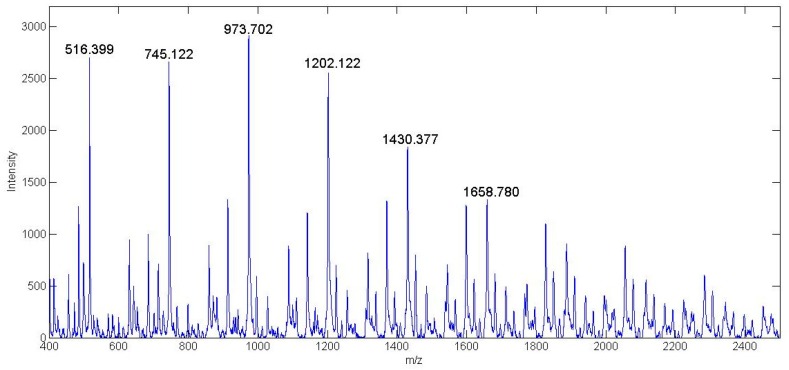
MALDI-TOF analysis of G1 HPAMAM.

**Figure 7:**
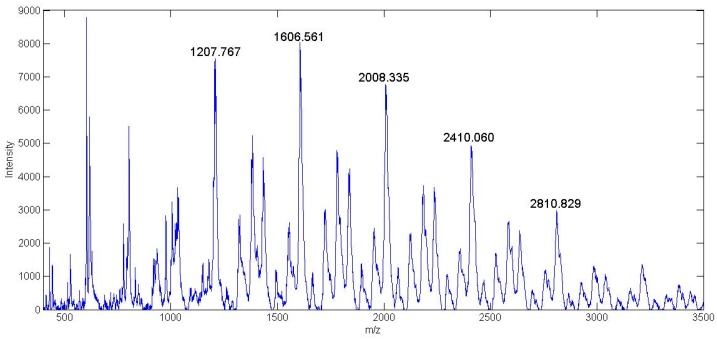
MALDI-TOF analysis of G1 NAP-HPAMAM.

Figure 6 and Figure 7 show the MALDI-TOF analysis done on the synthesized HPAMAM and NAP-HPAMAM compounds respectively. The matrix used for these experiments was α-cyano-4-hydroxycinnamic acid (CHCA). Since MALDI-TOF is a soft ionization mass spectroscopy technique, there is no fragmentation occurring during testing. This means that the entire range of peaks seen is a specific hyperbranched molecule with its own branching chemistry.

**Figure 8:**
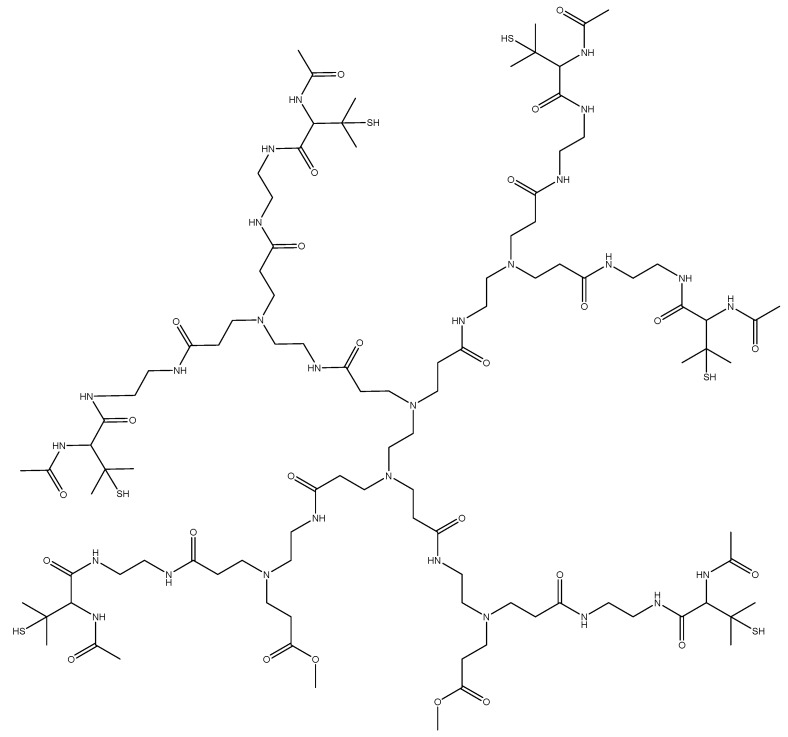
Structure of six-armed NAP-HPAMAM (M_W_ = 2413.14 Da).

A wide range of mass peaks was seen, as there are unreacted components still present as the synthesis process of the G1 HPAMAM progresses. The spectra of the materials are characteristic of polymers, where each increment signifies a repeat unit. The spectrum of HPAMAM shows the molecular weight of multiple different branched polymers. The four-armed HPAMAM starts at a mass of 516 Da and is increased by increments of 229 Da with the addition of another branched functional group. Mass peaks past 1430 Da, which is the G1 eight-armed HPAMAM, are most likely due to HPAMAM molecules attaching to each other. The second spectrum shows the attachment of NAP-thiolactone to free primary amine site branches in HPAMAM. The molecular weight of NAP-thiolactone is 173.23 Da, so the 229 Da mass increments between peaks seen in Figure 6 are increased to approximately 402 Da for each additional HPAMAM branch. An example of one of the NAP branched hyperbranched structures is shown in Figure 8. Each peak seen on both MALDI-TOF spectra can be used to construct some variation of the modified HPAMAM structure. The average molecular weight peak of the HPAMAM spectrum is seen at the 973 Da mark, which is the weight of a six-armed PAMAM structure. This is also confirmed by the ATTO-TAG FQ results which gave an estimated 6.40 primary amine sites per HPAMAM molecule.

## 4. Conclusions

SNAP-HPAMAM was able to show excellent stability and controllability when releasing NO, depending on what polymer matrix blend was made. The synthesis process is shown to be much less tedious than that of dendrimers while still maintaining a relatively high functionality of approximately 6.40 ± 0.309 primary amines per hyperbranched molecule. A large reservoir of NO of 1.901 ± 0.116 µmol of NO/mg was also demonstrated by the material, allowing SNAP-HPAMAM to modify a variety of polymers to be high-capacity NO-donating materials. These high-capacity NO-releasing polymers can then be applied to implantable devices and sensors to increase their longevity.

## References

[1] Chaux A., Min Ruanet X., Fishbein M.C., Ouyang Y., Kaul S., Pass J.A., Matloff J.M. (1998). Perivascular delivery of a nitric oxide donor inhibits neointimal hyperplasia in vein grafts implanted in the arterial circulation. J. Thorac. Cardiovasc. Surg..

[2] Gifford R. (2005). Mediation ofin vivo glucose sensor inflammatory response via nitric oxide release. J. Biomed. Mater. Res. Part A.

[3] Fang F.C. (1997). Perspectives series: Host/pathogen interactions. Mechanisms of nitric oxide-related antimicrobial activity. J. Clin. Investig..

[4] Williams D.L.H. (1999). The chemistry of S-nitrosothiols. Acc. Chem. Res..

[5] Keefer L.K. (1996). ONOates (1-substituted diazen-1-ium-1, 2-diolates) as nitric oxide donors: Convenient nitric oxide dosage forms. Methods Enzym..

[6] Wang P.G. (2002). Nitric oxide donors: Chemical activities and biological applications. Chem. Rev..

[7] Buhleier E. (1978). Ligand Structure and Complexation, XIII: 2,2’-Bipyridine as a Building Block for New Aza Crown Ethers and Cryptands. Chemsche Ber..

[8] Newkome G.R. (1985). Micelles. Part 1. Cascade molecules: A new approach to micelles. A [27]-arborol. J. Org. Chem..

[9] Tomalia D.A. (1985). A new class of polymers: Starburst-dendritic macromolecules. Polym. J..

[10] Gillies E.R., Frechet J.M. (2005). Design, synthesis, and biological evaluation of a robust, biodegradable dendrimer. Drug Discov. Today.

[11] Majoros I.J. (2006). PAMAM dendrimer-based multifunctional conjugate for cancer therapy: Synthesis, characterization, and functionality. Biomacromolecules.

[12] Han S.-O. (2000). Development of biomaterials for gene therapy. Mol. Ther..

[13] Kukowska-Latallo J.F. (1996). Efficient transfer of genetic material into mammalian cells using Starburst polyamidoamine dendrimers. Proc. Natl. Acad. Sci. USA.

[14] Duncan R., Izzo L. (2005). Dendrimer biocompatibility and toxicity. Adv. Drug Del. Rev..

[15] Nyitrai G. (2013). Sodium selective ion channel formation in living cell membranes by polyamidoamine dendrimer. Biochim. Biophys. Acta (Bba) Biomembr..

[16] Johnson T.A. (2010). Reduced ischemia/reperfusion injury via glutathione-initiated nitric oxide-releasing dendrimers. Nitric Oxide.

[17] Stasko N.A. (2008). S-Nitrosothiol-Modified Dendrimers as Nitric Oxide Delivery Vehicles. Biomacromolecules.

[18] Lu Y. (2013). Nitric oxide-releasing amphiphilic poly (amidoamine) (PAMAM) dendrimers as antibacterial agents. Biomacromolecules.

[19] Worley B.V. (2016). Active release of nitric oxide-releasing dendrimers from electrospun polyurethane fibers. Acs Biomater. Sci. Eng..

[20] Frechet J.M. (1995). Self-condensing vinyl polymerization: An approach to dendritic materials. Science.

[21] Kou Y., Wan A. (2008). Synthesis of novel N-diazeniumdiolates based on hyperbranched polyethers. Bioorg. Med. Chem. Lett..

[22] Keefer L.K. (2001). Chemistry of the Diazeniumdiolates I. Structural and Spectral Characteristics of the [N (O) NO]−Functional Group. Nitric Oxide.

[23] Klaykruayat B. (2010). Chemical modification of chitosan with cationic hyperbranched dendritic polyamidoamine and its antimicrobial activity on cotton fabric. Carbohydr. Polym..

[24] Moynihan H.A., Roberts S.M. (1994). Preparation of some novel S-nitroso compounds as potential slow-release agents of nitric oxide in vivo. J. Chem. Soc. Perkin Trans. 1.

[25] Yang B.K. (2003). Nitrite reduction to nitric oxide by deoxyhemoglobin vasodilates the human circulation. Free Radic. Res..

[26] Lu Y. (2011). Fabrication of nitric oxide-releasing porous polyurethane membranes-coated needle-type implantable glucose biosensors. Chem. Mater..

[27] Sun B. (2012). Nitric oxide-releasing dendrimers as antibacterial agents. Biomacromolecules.

[28] Yates C., Hayes W. (2004). Synthesis and applications of hyperbranched polymers. Eur. Polym. J..

[29] Mikhelson K. (1994). Ion-selective electrodes in PVC matrix. Sens. Actuators B Chem..

[30] Gierke G.E. (2016). S-Nitroso-N-acetyl-D-penicillamine covalently linked to polydimethylsiloxane (SNAP–PDMS) for use as a controlled photoinitiated nitric oxide release polymer. Sci. Technol. Adv. Mat..

[31] Hopkins S.P., Frost M.C. (2018). Synthesis and Characterization of Controlled Nitric Oxide Release from S- Nitroso-N-Acetyl-D-Penicillamine Covalently Linked to Polyvinyl Chloride (SNAP-PVC). Bioengineering.

[32] Frost M.C., Meyerhoff M.E. (2005). Synthesis, characterization, and controlled nitric oxide release from S-nitrosothiol-derivatized fumed silica polymer filler particles. J. Biomed. Mater. Res. Part A.

[33] He W., Frost M.C. (2016). CellNO trap: Novel device for quantitative, real-time, direct measurement of nitric oxide from cultured RAW 267.4 macrophages. Redox Biol..

[34] Lindström A., Hakkarainen M. (2006). Environmentally friendly plasticizers for poly(vinyl chloride)—Improved mechanical properties and compatibility by using branched poly(butylene adipate) as a polymeric plasticize. J. Appl. Polym. Sci..

[35] Liu J. (1991). Design of 3-(4-carboxybenzoyl)-2-quinolinecarboxaldehyde as a reagent for ultrasensitive determination of primary amines by capillary electrophoresis using laser fluorescence detection. Anal. Chem..

[36] Ellman G.L. (1959). Tissue sulfhydryl groups. Arch. Biochem. Biophys..

